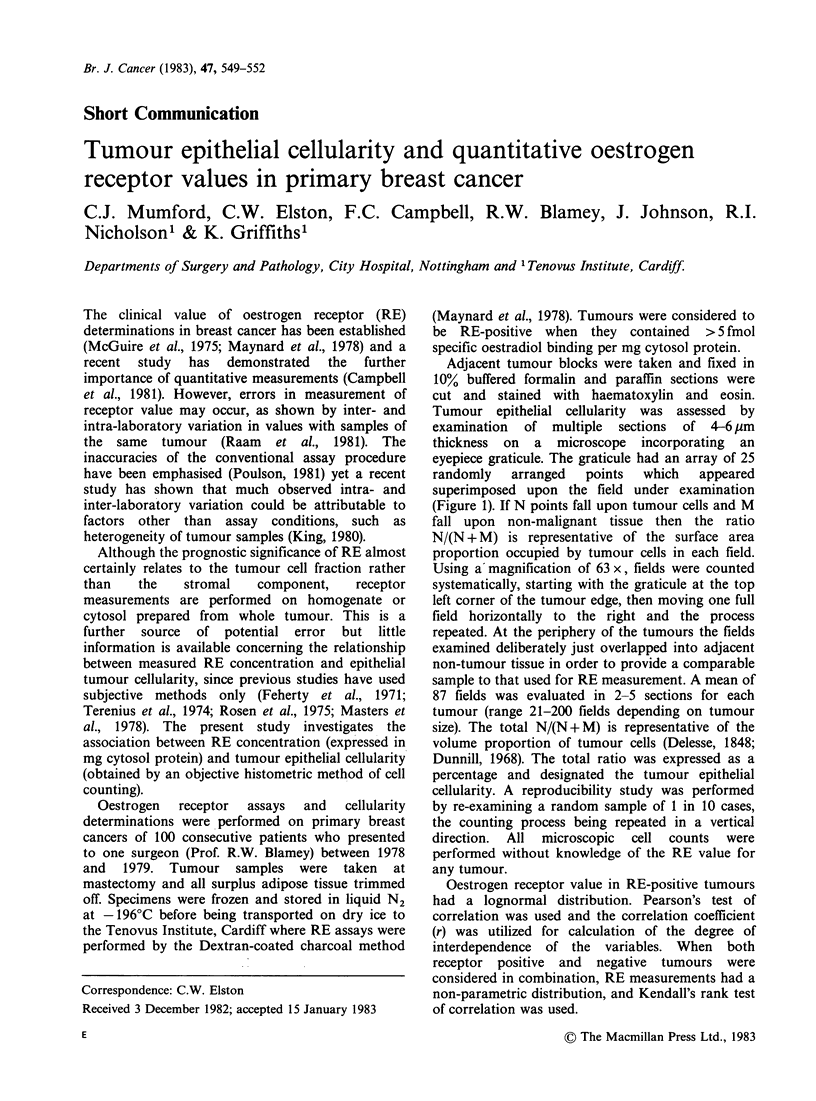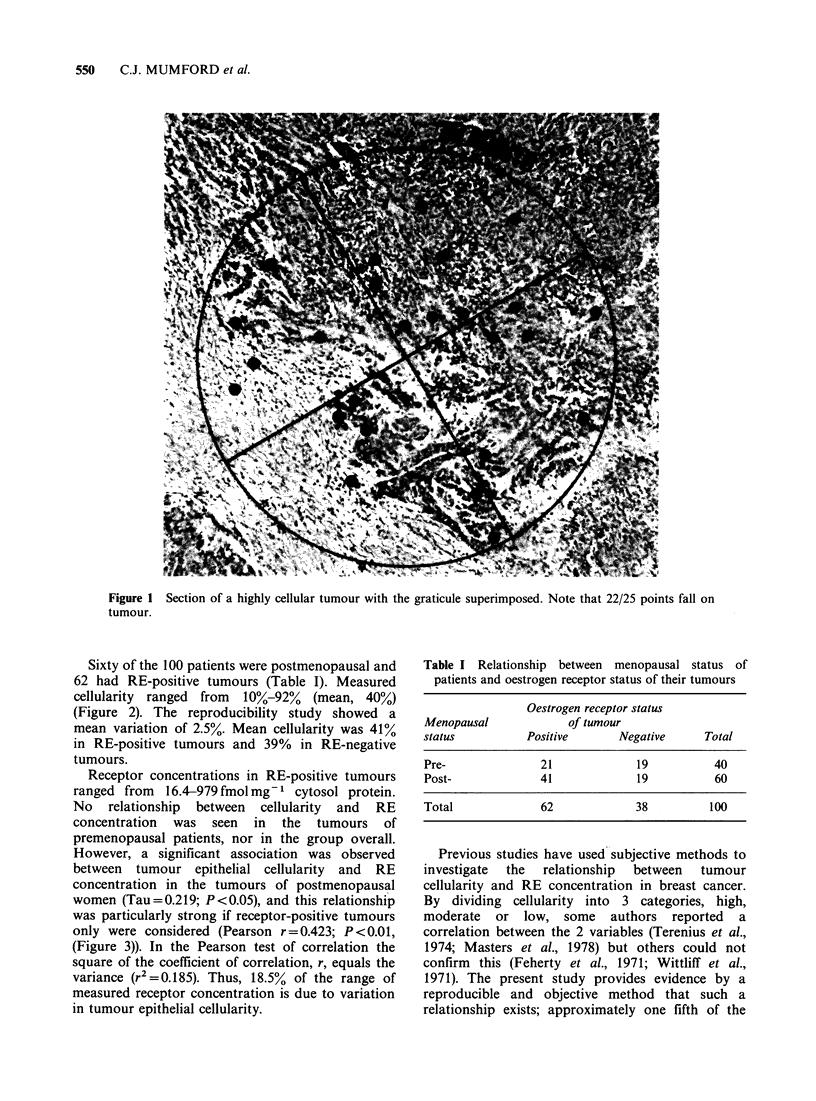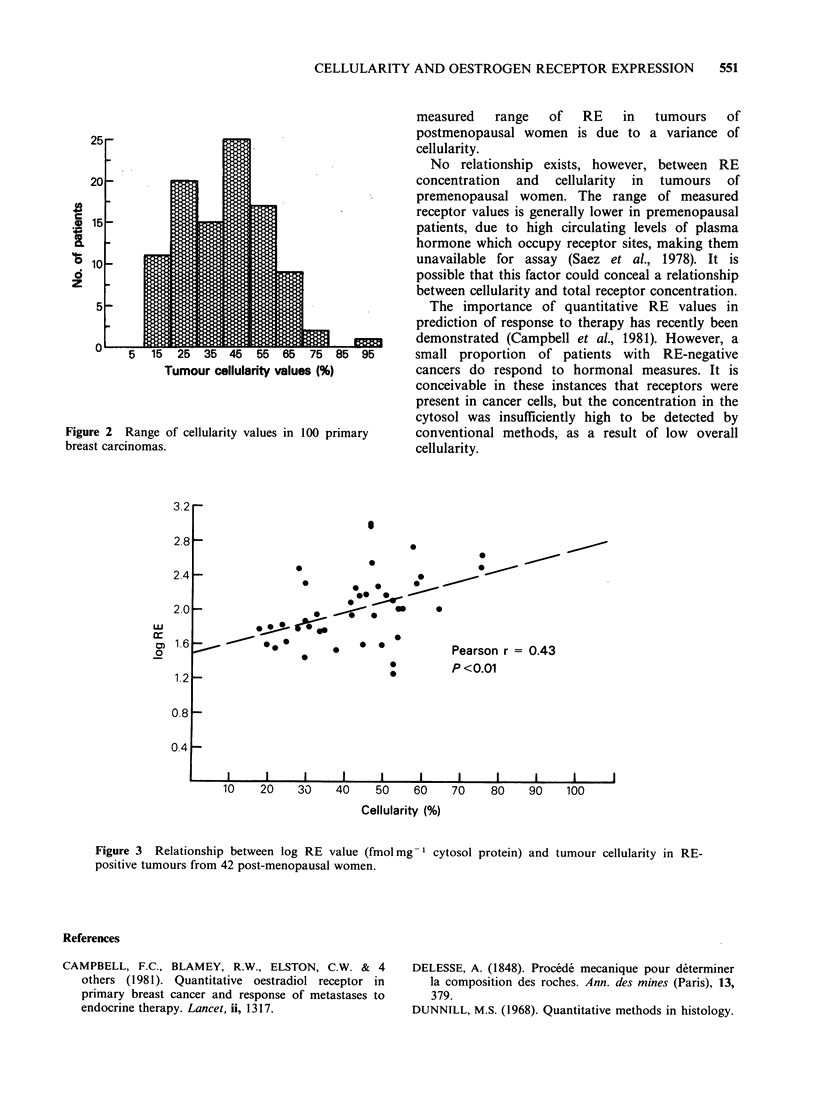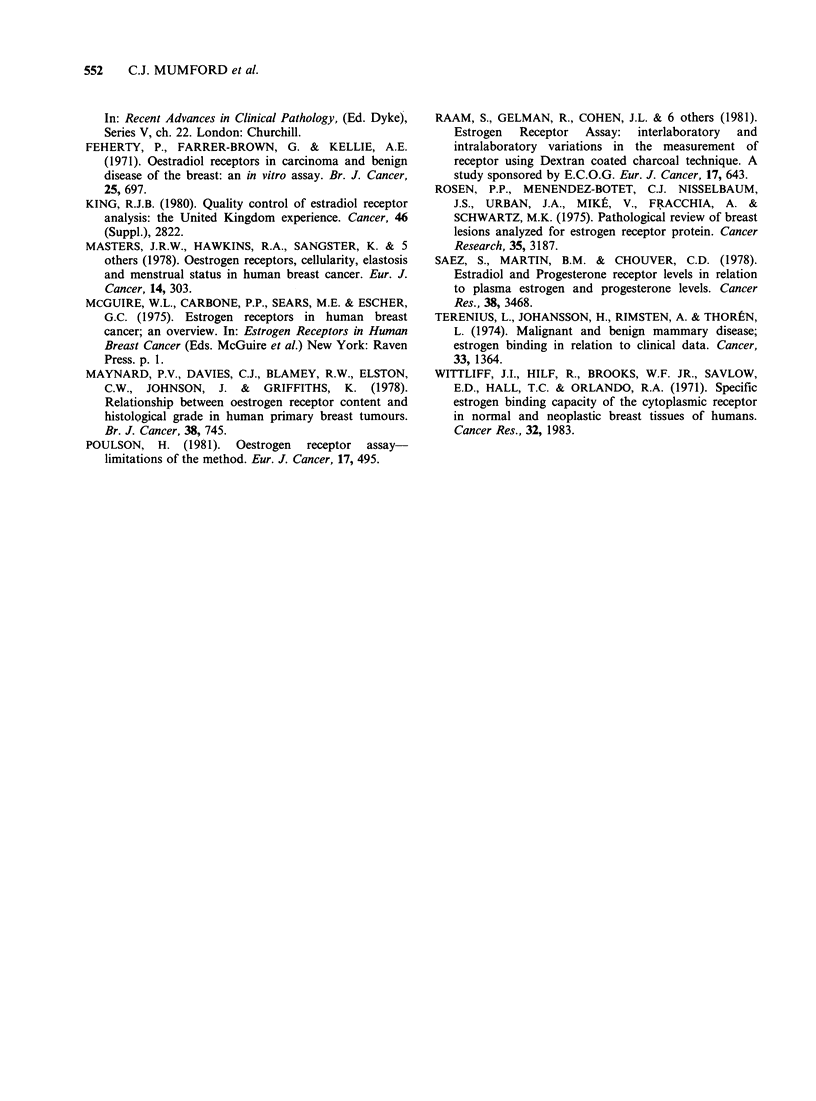# Tumour epithelial cellularity and quantitative oestrogen receptor values in primary breast cancer.

**DOI:** 10.1038/bjc.1983.87

**Published:** 1983-04

**Authors:** C. J. Mumford, C. W. Elston, F. C. Campbell, R. W. Blamey, J. Johnson, R. I. Nicholson, K. Griffiths

## Abstract

**Images:**


*Br. J. Cancer* (1983), **47**, 549–552

### Short Communication

### Tumour epithelial cellularity and quantitative oestrogen receptor values in primary breast cancer

C.J. Mumford, C.W. Elston, F.C. Campbell, R.W. Blamey, J. Johnson, R.I. Nicholson[1] & K. Griffiths[1]

*Departments of Surgery and Pathology, City Hospital, Nottingham and [1]Tenovus Institute, Cardiff.*

The clinical value of oestrogen receptor (RE) determinations in breast cancer has been established (McGuire *et al.*, 1975; Maynard *et al.*, 1978) and a recent study has demonstrated the further importance of quantitative measurements (Campbell *et al.*, 1981). However, errors in measurement of receptor value may occur, as shown by inter- and intra-laboratory variation in values with samples of the same tumour (Raam *et al.*, 1981). The inaccuracies of the conventional assay procedure have been emphasised (Poulson, 1981) yet a recent study has shown that much observed intra- and inter-laboratory variation could be attributable to factors other than assay conditions, such as heterogeneity of tumour samples (King, 1980).

Although the prognostic significance of RE almost certainly relates to the tumour cell fraction rather than the stromal component, receptor measurements are performed on homogenate or cytosol prepared from whole tumour. This is a further source of potential error but little information is available concerning the relationship between measured RE concentration and epithelial tumour cellularity, since previous studies have used subjective methods only (Feherty *et al.*, 1971; Terenius *et al.*, 1974; Rosen *et al.*, 1975; Masters *et al.*, 1978). The present study investigates the association between RE concentration (expressed in mg cytosol protein) and tumour epithelial cellularity (obtained by an objective histometric method of cell counting).

Oestrogen receptor assays and cellularity determinations were performed on primary breast cancers of 100 consecutive patients who presented to one surgeon (Prof. R.W. Blamey) between 1978 and 1979. Tumour samples were taken at mastectomy and all surplus adipose tissue trimmed off. Specimens were frozen and stored in liquid $N_2$ at $-196$°C before being transported on dry ice to the Tenovus Institute, Cardiff where RE assays were performed by the Dextran-coated charcoal method (Maynard *et al.*, 1978). Tumours were considered to be RE-positive when they contained >5 fmol specific oestradiol binding per mg cytosol protein.

Adjacent tumour blocks were taken and fixed in 10% buffered formalin and paraffin sections were cut and stained with haematoxylin and eosin. Tumour epithelial cellularity was assessed by examination of multiple sections of 4–6 $\mu$m thickness on a microscope incorporating an eyepiece graticule. The graticule had an array of 25 randomly arranged points which appeared superimposed upon the field under examination (Figure 1). If N points fall upon tumour cells and M fall upon non-malignant tissue then the ratio N/(N+M) is representative of the surface area proportion occupied by tumour cells in each field. Using a magnification of 63×, fields were counted systematically, starting with the graticule at the top left corner of the tumour edge, then moving one full field horizontally to the right and the process repeated. At the periphery of the tumours the fields examined deliberately just overlapped into adjacent non-tumour tissue in order to provide a comparable sample to that used for RE measurement. A mean of 87 fields was evaluated in 2–5 sections for each tumour (range 21–200 fields depending on tumour size). The total N/(N+M) is representative of the volume proportion of tumour cells (Delesse, 1848; Dunnill, 1968). The total ratio was expressed as a percentage and designated the tumour epithelial cellularity. A reproducibility study was performed by re-examining a random sample of 1 in 10 cases, the counting process being repeated in a vertical direction. All microscopic cell counts were performed without knowledge of the RE value for any tumour.

Oestrogen receptor value in RE-positive tumours had a lognormal distribution. Pearson's test of correlation was used and the correlation coefficient (*r*) was utilized for calculation of the degree of interdependence of the variables. When both receptor positive and negative tumours were considered in combination, RE measurements had a non-parametric distribution, and Kendall's rank test of correlation was used.

Correspondence: C.W. Elston

Received 3 December 1982; accepted 15 January 1983

© The Macmillan Press Ltd., 1983

**Figure 1** Section of a highly cellular tumour with the graticule superimposed. Note that 22/25 points fall on tumour.

Sixty of the 100 patients were postmenopausal and 62 had RE-positive tumours (Table I). Measured cellularity ranged from 10%–92% (mean, 40%) (Figure 2). The reproducibility study showed a mean variation of 2.5%. Mean cellularity was 41% in RE-positive tumours and 39% in RE-negative tumours.

Receptor concentrations in RE-positive tumours ranged from 16.4–979 fmol mg$^{-1}$ cytosol protein. No relationship between cellularity and RE concentration was seen in the tumours of premenopausal patients, nor in the group overall. However, a significant association was observed between tumour epithelial cellularity and RE concentration in the tumours of postmenopausal women (Tau = 0.219; $P<0.05$), and this relationship was particularly strong if receptor-positive tumours only were considered (Pearson $r=0.423$; $P<0.01$, (Figure 3)). In the Pearson test of correlation the square of the coefficient of correlation, $r$, equals the variance ($r^2=0.185$). Thus, 18.5% of the range of measured receptor concentration is due to variation in tumour epithelial cellularity.

**Table I** Relationship between menopausal status of patients and oestrogen receptor status of their tumours

| *Menopausal status* | *Oestrogen receptor status of tumour* *Positive* | *Negative* | *Total* |
|---|---|---|---|
| Pre- | 21 | 19 | 40 |
| Post- | 41 | 19 | 60 |
| Total | 62 | 38 | 100 |

Previous studies have used subjective methods to investigate the relationship between tumour cellularity and RE concentration in breast cancer. By dividing cellularity into 3 categories, high, moderate or low, some authors reported a correlation between the 2 variables (Terenius *et al.*, 1974; Masters *et al.*, 1978) but others could not confirm this (Feherty *et al.*, 1971; Wittliff *et al.*, 1971). The present study provides evidence by a reproducible and objective method that such a relationship exists; approximately one fifth of the

**Figure 2** Range of cellularity values in 100 primary breast carcinomas.

measured range of RE in tumours of postmenopausal women is due to a variance of cellularity.

No relationship exists, however, between RE concentration and cellularity in tumours of premenopausal women. The range of measured receptor values is generally lower in premenopausal patients, due to high circulating levels of plasma hormone which occupy receptor sites, making them unavailable for assay (Saez *et al.*, 1978). It is possible that this factor could conceal a relationship between cellularity and total receptor concentration.

The importance of quantitative RE values in prediction of response to therapy has recently been demonstrated (Campbell *et al.*, 1981). However, a small proportion of patients with RE-negative cancers do respond to hormonal measures. It is conceivable in these instances that receptors were present in cancer cells, but the concentration in the cytosol was insufficiently high to be detected by conventional methods, as a result of low overall cellularity.

**Figure 3** Relationship between log RE value (fmol mg$^{-1}$ cytosol protein) and tumour cellularity in RE-positive tumours from 42 post-menopausal women.

### References

CAMPBELL, F.C., BLAMEY, R.W., ELSTON, C.W. & 4 others (1981). Quantitative oestradiol receptor in primary breast cancer and response of metastases to endocrine therapy. *Lancet*, **ii**, 1317.

DELESSE, A. (1848). Procédé mecanique pour déterminer la composition des roches. *Ann. des mines* (Paris), **13**, 379.

DUNNILL, M.S. (1968). Quantitative methods in histology.

In: *Recent Advances in Clinical Pathology,* (Ed. Dyke), Series V, ch. 22. London: Churchill.

FEHERTY, P., FARRER-BROWN, G. & KELLIE, A.E. (1971). Oestradiol receptors in carcinoma and benign disease of the breast: an *in vitro* assay. *Br. J. Cancer,* **25,** 697.

KING, R.J.B. (1980). Quality control of estradiol receptor analysis: the United Kingdom experience. *Cancer,* **46** (Suppl.), 2822.

MASTERS, J.R.W., HAWKINS, R.A., SANGSTER, K. & 5 others (1978). Oestrogen receptors, cellularity, elastosis and menstrual status in human breast cancer. *Eur. J. Cancer,* **14,** 303.

MCGUIRE, W.L., CARBONE, P.P., SEARS, M.E. & ESCHER, G.C. (1975). Estrogen receptors in human breast cancer; an overview. In: *Estrogen Receptors in Human Breast Cancer* (Eds. McGuire *et al.*) New York: Raven Press. p. 1.

MAYNARD, P.V., DAVIES, C.J., BLAMEY, R.W., ELSTON, C.W., JOHNSON, J. & GRIFFITHS, K. (1978). Relationship between oestrogen receptor content and histological grade in human primary breast tumours. *Br. J. Cancer,* **38,** 745.

POULSON, H. (1981). Oestrogen receptor assay—limitations of the method. *Eur. J. Cancer,* **17,** 495.

RAAM, S., GELMAN, R., COHEN, J.L. & 6 others (1981). Estrogen Receptor Assay: interlaboratory and intralaboratory variations in the measurement of receptor using Dextran coated charcoal technique. A study sponsored by E.C.O.G. *Eur. J. Cancer,* **17,** 643.

ROSEN, P.P., MENENDEZ-BOTET, C.J. NISSELBAUM, J.S., URBAN, J.A., MIKÉ, V., FRACCHIA, A. & SCHWARTZ, M.K. (1975). Pathological review of breast lesions analyzed for estrogen receptor protein. *Cancer Research,* **35,** 3187.

SAEZ, S., MARTIN, B.M. & CHOUVER, C.D. (1978). Estradiol and Progesterone receptor levels in relation to plasma estrogen and progesterone levels. *Cancer Res.,* **38,** 3468.

TERENIUS, L., JOHANSSON, H., RIMSTEN, A. & THORÉN, L. (1974). Malignant and benign mammary disease; estrogen binding in relation to clinical data. *Cancer,* **33,** 1364.

WITTLIFF, J.I., HILF, R., BROOKS, W.F. JR., SAVLOW, E.D., HALL, T.C. & ORLANDO, R.A. (1971). Specific estrogen binding capacity of the cytoplasmic receptor in normal and neoplastic breast tissues of humans. *Cancer Res.,* **32,** 1983.